# Supplementary material for: Unveiling the Genome of the Diploid Wild Sugarcane Relative Narenga porphyrocoma (Hance) Bor
Source: Int J Mol Sci. 2025 Jun 26;26(13):6124. doi: 10.3390/ijms26136124 (PMC12249692; doi:10.3390/ijms26136124)
Supplement: Supplementary file 1 [file ijms-26-06124-s001.zip › ijms-3553143-supplementary.pdf]

Table S1. Summary statistics of sequencing data

Sequencing	Data type	Total reads	Total base (GB)	Max length (bp)	Mean length (bp)	N50 length (bp)
**HiFi**	Subreads	60,861,825	1,023.75	562,647	16,830	18,566
	CCS reads	3,926,609	73.60	49,959	18,752	18,609
**Hi-C**	Raw data	2,747,483,708	425.86	/	/	/
	Clean data	2,346,801,496	363.43	/	/	/

Table S2. Functional annotation statistics of predicted protein-coding genes

	Number	Percentage of genes
Total gene	70,680	-
Nr	69,713	98.63%
Swissprot	48,125	68.09%
KEGG	46,410	65.66%
KOG	44,350	62.75%
TrEMBL	68,361	96.72%
Interpro	57,467	81.31%
GO	34,386	48.65%
Overall annotated	70,033	99.08%

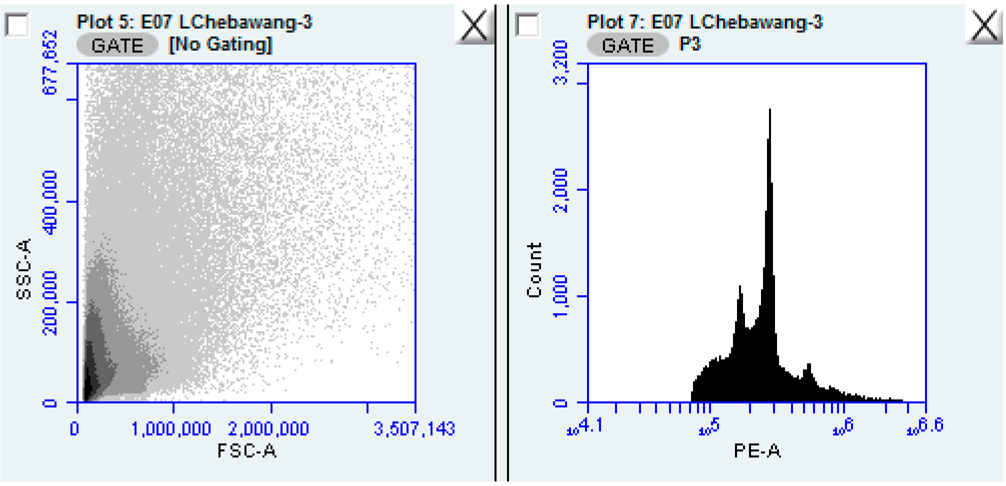

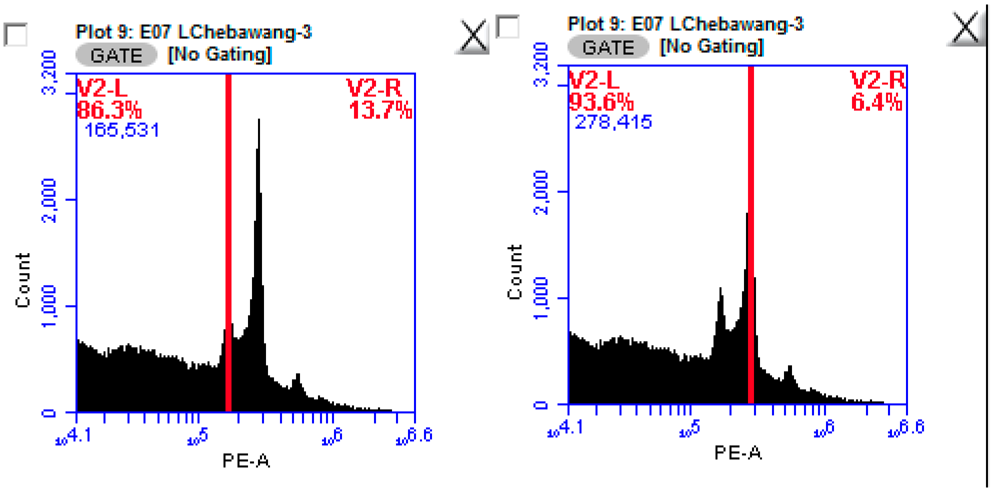

Figure S1. Flow cytometry analysis confirmed that *N. porphyrocoma* (Hance) Bor was diploid.

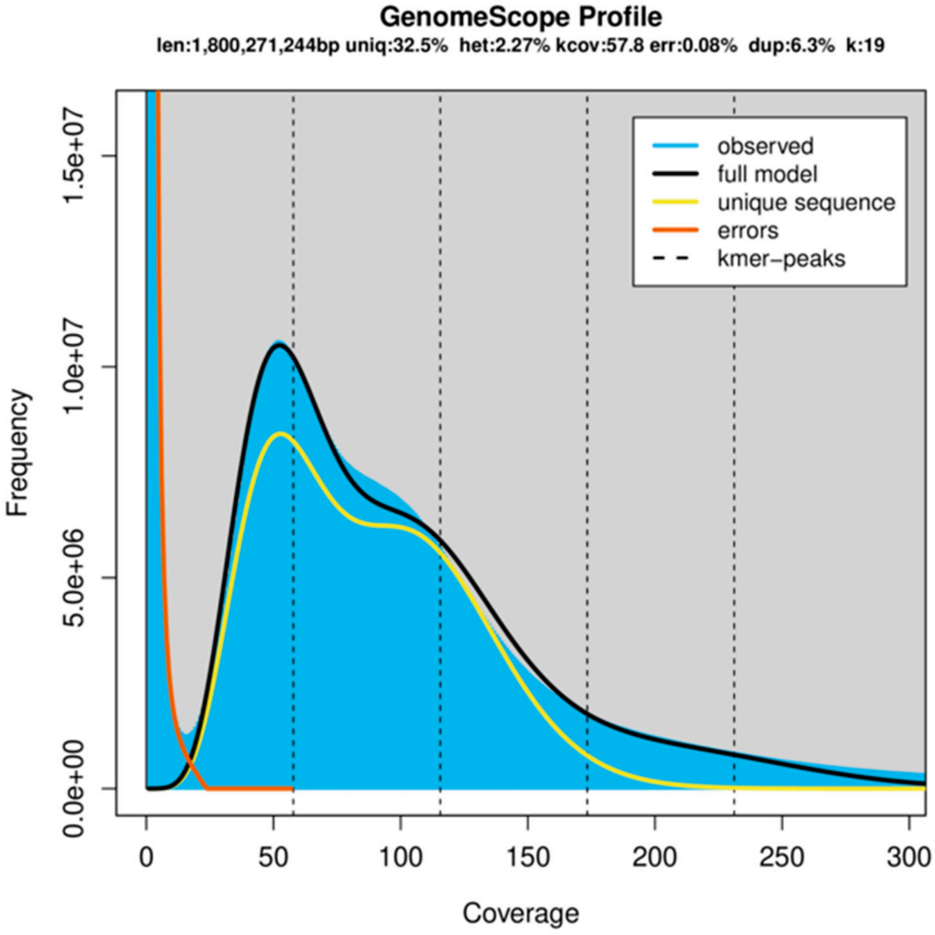

Figure S2. Genome survey based on K-mer analysis.
